# Retrospective Lineage Tracing: An Optimal Approach for the Study of Intrinsic Cellular Development

**DOI:** 10.7759/cureus.82241

**Published:** 2025-04-14

**Authors:** Alessandro Hammond, Anuj Mankad

**Affiliations:** 1 Hematology and Oncology, Massachusetts General Hospital, Boston, USA; 2 Hematology and Medical Oncology, Oregon Health and Science University School of Medicine, Portland, USA

**Keywords:** clonal hematopoiesis of indeterminate potential, extra-medullary hematopoiesis, lineage tracing, models of thrombosis and hemostasis, preoperative hematopoiesis

## Abstract

Lineage tracing is an essential tool for understanding cellular development and tissue dynamics. This review examines retrospective lineage tracing as an optimal approach for studying cellular development and contrasts retrospective with prospective lineage tracing methods. Retrospective lineage tracing approaches leverage naturally occurring genetic barcodes, such as single nucleotide polymorphisms (SNPs), copy number variants (CNVs), and mitochondrial DNA mutations, which enables the detailed reconstruction of cell lineages without prior genetic manipulation. Researchers can ultimately infer developmental trajectories and clonal relationships across hematopoiesis and tumorigenesis by analyzing these endogenous markers. This paper considers how retrospective lineage tracing methods circumvent the limitations of prospective approaches, such as the need for exogenous labeling, and is valuable for studying human hematopoiesis.

## Introduction and background

Lineage tracing is the process of understanding and determining the emergence of all cells in a tissue, organ, or organism by following the cells as they proliferate and differentiate from progenitors to the final phase of development of the progeny. This approach is fundamental to understanding developmental biology, stem cell function, and disease progression. The two major paradigms of lineage tracing are prospective and retrospective lineage tracing. In a prospective approach, exogenous markers are introduced and then tracked forward in time as labeled cells generate progeny. This was the original form of lineage tracing used for hematopoietic stem cells, embryonic development, and cancer studies. Historically, prospective methods involved creating chimeric embryos or organisms through cell engraftment between species or injecting colored/fluorescent dyes into originator cells [1,2]. In contrast, retrospective lineage tracing uses endogenous markers to trace progeny back to their originator cells, allowing researchers to reconstruct developmental histories after they have occurred without experimental intervention during development.

Comparing prospective and retrospective approaches

Prospective lineage tracing utilizes exogenously introduced genetic labels or barcodes deliberately inserted into cells before tracking their progeny. This approach enables simultaneous studies of lineage and cell type but has several limitations: it requires genetic manipulation of model animals or cultured cells [2,3], necessitates prior knowledge of the initial cell state [1], cannot be employed before the developmental stage at which labels are introduced, and is impractical for human patient tissues [4]. Modern prospective lineage tracing often combines CRISPR/Cas9 or DNA recombination with next-generation sequencing to determine cell states of progeny [1]. Homoplasy - when similar markings appear in different lineage branches - can confuse the origins of descendant cells, particularly with one-time genetic changes using CRISPR-Cas9 or recombinase methods. Thus, ideally, prospective lineage tracing would uniquely label each cell to provide information on clonal heterogeneity [1]. However, even this may be limiting, since using a single barcode limits resolution, even when leveraging variation in barcode insertion location [1], and does not account for dynamic changes in cell state. This can be achieved through evolving barcode systems, such as the DNA Typewriter procedure [5-8], where Cas9 endonuclease combined with various single guide RNAs introduces new barcodes under different conditions. However, this requires continuous CRISPR-Cas9 activity, which has been associated with cellular lethality, lineage death, or developmental issues, potentially limiting barcode insertions and resolution. Multiple DNA double-strand breaks may also result in the loss of previous barcodes, erasing developmental history [1]. Alternative approaches using genetic recombination (PolyLox system) or base-editing enzymes avoid double-strand breaks but introduce other complications, including off-target recombination or unintended mutations affecting cell growth and differentiation. Regardless, the issues inherent in introducing exogenous barcodes and the inability to trace cells prior to the introduction of these barcodes are limitations of the prospective approach.

In contrast, retrospective lineage tracing leverages naturally occurring cellular mutations that accumulate over time as inherent barcodes and thereby avoids many of these limitations by allowing for tracing cells back to the very first source cell without requiring prior knowledge of the initial cell state. Importantly, retrospective lineage tracing doesn't require exogenous genome alterations that could have unexpected consequences and allows analysis of any purifiable cell populations. This approach generally prevents homoplasy and allows more precise differentiation of clonally-derived lineages, making it particularly suitable for higher-resolution fate mapping of human tissues compared to even dynamic prospective methods. Furthermore, since it maps lineage relationships by analyzing backward in time, it allows researchers to distinguish early and late fate decisions by constructing phylogenetic trees. One disadvantage of retrospective lineage tracing is that it can be lower throughput and more expensive. This issue may be partly solved via recent advances in high-throughput sequencing that have enhanced the detection of naturally occurring genetic variations as barcodes [3,5], making retrospective lineage tracing more cost-effective.

Evolution of lineage tracing methods: low-resolution approaches

Early lineage tracing methods had significant limitations. Techniques using fluorescent dyes or chimeric systems generally only allowed the determination of the combined fate of a cell population rather than tracking at the single-cell level [3,4]. These population-level experiments could not account for heterogeneity within labeled populations and sometimes accidentally included non-target cells. Such approaches risked confounding the activity of a single cell type performing multiple functions with that of multiple cell types (each performing unique functions) arising from the same progenitors.

High-resolution approaches

To overcome these limitations, modern lineage tracing employs genetic barcoding strategies. These involve using heritable markers inserted into, or leveraging existing distinguishable DNA sequence differences in, the genome to identify clonal descendants. High-throughput sequencing now permits the precise identification of cell types and their relationships [4,5]. Cellular barcodes - nucleic acid sequences that track individual cells spatially and temporally - can be synthetically produced DNA sequences, induced insertions, deletions, or recombinations in DNA sequences, or naturally occurring nuclear or mitochondrial mutations [6-8]. These barcoding approaches permit the tracking of progenitor clones through reproduction and differentiation while recording specific state transitions and other important biological events. For example, current prospective lineage tracing technologies apply CRISPR-Cas9 to mark individual cells with insertions or deletions and later identify the developmental lineage of clonal descendants. Unlike fluorescent reporters, which have inherent limitations on the number of trackable clones based on available color combinations, DNA barcodes can be varied in length and sequence to theoretically record every cell division event in an organism, with the recorded information read at the experiment's conclusion using high-throughput sequencing.

## Review

Main text

**Figure 1:**
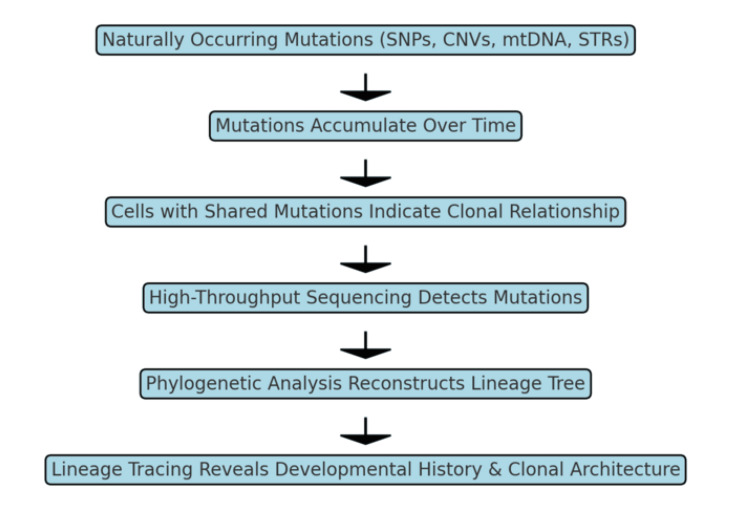
Retrospective Lineage Tracing SNPs: single nucleotide polymorphisms; CNVs: copy number variants; mtDNA: mitochondrial DNA; STRs: short tandem repeats Image created by the author. Text content adapted from Reference [5].

When combined with multimodal, single-cell-resolution measurements, retrospective lineage tracing can reveal the dynamics of both healthy and diseased cells (Figure 1). By analyzing endogenous genetic markers alongside gene expression profiles or chromatin accessibility patterns, researchers can determine the overall lineage tree and the status of each cell within a tissue after differentiation [1,5]. This approach offers inherent advantages: cells don't require pre-labeling, enabling analysis of human patient samples (including cancers and archived clinical specimens) as well as non-model organisms or situations where initial experimental manipulation is impossible [1,2,6,7].

Next-generation sequencing has made lineage tracing using naturally occurring mutations relatively straightforward [4]. Somatic variants function as genetic barcodes similar to CRISPR-Cas9-induced alterations, automatically passing to cell progeny [4]. As mutations accumulate over time, researchers can infer the entire trajectory from progenitor to differentiated cell, including intermediate sub-clones [4].

Multimodal single-cell analysis in retrospective lineage tracing

Both prospective and retrospective lineage tracing have been employed to delineate cell development hierarchies in various tissues by combining lineage analysis with measurements of transcriptional or chromatin states. These approaches have been extensively applied to hematopoiesis research, clarifying the contributions of hematopoietic stem and progenitor cells (HSPCs) under normal and stress conditions. However, studying HSPC contributions to human hematopoiesis specifically requires retrospective lineage tracing [5].

Similarly, only retrospective lineage tracing of human HSPCs can identify cancer-initiating cells, detect heterogeneity in chemotherapy-resistant clones, and reveal critical information about myelodysplastic syndrome and leukemia development. By using copy number abnormalities, point mutations, and structural alterations as barcodes, researchers can study cancer clonal architecture and its temporal changes [9]. Intratumor functional heterogeneity, a key determinant in cancer progression and therapy success, can only be fully understood through retrospective lineage approaches [9]. Endogenous barcoding is ideal for understanding clonal outgrowth with different tumor initiation, therapy resistance, and metastasis potential. This approach can help uncover founding cells, determine cancer origination timing, and evaluate clonal growth rates to inform treatment timelines. Combined with transcriptional and chromatin state analysis of subclones, this could enable targeted therapies against the most aggressive cancer subpopulations.

The Tracking Non-small Cell Lung Cancer Evolution through Therapy (TRACERx) study exemplifies this approach, using genetic analysis of patient samples to delineate lung cancer evolution and correlate tumor clonal heterogeneity with therapeutic outcomes. Phylogenetic trees based on sequence variants revealed the timing of genetic mutations and heterogeneous carcinogenic mechanisms, providing insights into the relationship between mutations and chromosomal instability [9]. Similar studies investigating metastatic cancers and the effects of therapeutic interventions on cancer cell evolutionary trajectories could lead to novel anti-cancer therapies [10].

Endogenous barcode sources for retrospective lineage tracing

Clonal expansion, the outgrowth of cell populations from individual stem cells, occurs in multiple human tissues, including blood, skin, epithelia, liver, colon, pancreas, endometrium, prostate, and brain. Some inflammatory disorders also result in clonal expansion [5,7]. Retrospective lineage tracing has been applied beyond hematopoiesis and cancers to analyze mutational landscapes and clonal expansion in colonic epithelial cells during inflammatory bowel diseases, chronic liver conditions, and other tissue disorders [10]. These studies required retrospective approaches since clonal expansion had already occurred. Research on colonic mucosa has indicated that clonal growth may cause or perpetuate inflammatory bowel diseases, similar to how clonal hematopoiesis associates with leukemia and coronary heart disease [9,11]. Understanding clonal expansion in disease precursor states enables prevention, earlier detection, or treatment, but requires retrospective methods to analyze tissues after formation or disease progression [5,9].

For effective retrospective lineage tracing at single-cell resolution, researchers can use inheritable somatic mutations as barcodes [1]. Several sources of endogenous barcoding exist, each with distinct resolution capabilities and applications. Nuclear or mitochondrial genome sequencing changes represent one important source; Chapman et al. (2021) used somatic mutations accumulated in fetal HSPCs by 18 weeks post-conception to time the bifurcation of embryonic tissues and approximate blood cell progenitor quantities throughout development [7,12]. Polymorphic loci comprise another source, including transposable elements such as long interspersed nuclear elements (LINEs, including L1 retrotransposons) and short interspersed nuclear elements (SINEs, including Alu elements); single nucleotide polymorphisms (SNPs); short tandem repeats (STRs); and copy number variants (CNVs) [2-4].

CNVs are readily detectable for single-cell retrospective lineage tracing without requiring deep sequencing with high coverage [4], though CNVs are rare in healthy tissue, despite being found in skin and brain cells. While most CNVs are unique to specific cells, small groups sharing CNVs indicate clonal derivation, allowing only low-resolution phylogeny generation in healthy tissues [4]; their limited frequency in normal tissues restricts complete developmental phylogenetic reconstruction. In contrast, CNVs are abundant in cancer and change significantly during tumor progression, making them valuable for cancer lineage tracing. SNPs and small insertions/deletions, typically in non-coding genomic regions, are commonly used for healthy tissue lineage tracing. SNPs' reliable duplication during DNA replication has enabled tumor phylogenetic tree construction from both bulk DNA and single-cell whole genome/exome sequencing, though the latter presents challenges due to sparse SNP distribution, error-prone amplification, and non-uniform coverage [4]. Targeted sequencing of specific genomic regions containing key SNPs can mitigate these issues, allowing more cells to be sequenced with greater coverage at a lower cost [4]. L1 LINE retrotransposons, comprising up to 17% of the human genome, can serve as barcodes when mobile elements excise and reinsert into new genomic locations during cell division. This is particularly relevant in neural tissues, though transposition rates are low (less than one insertion per neuron). The small fraction of mobile LINEs can mark specific cellular subpopulations based on their infrequent relocation in founder cells [3,4].

Microsatellites as high-resolution lineage barcodes

Among the various endogenous barcode options, STRs offer unique advantages for high-resolution retrospective lineage tracing, including their known genomic locations [4]. They are the most prevalent endogenous barcode source, contributing the most de novo mutations with predictable changes dependent on repeat type and number. Even a small subset of STRs loci can serve as excellent barcodes for distinguishing cellular lineages.

Current methods, such as duplex molecular inversion probes developed by Tao et al., can capture over 50,000 microsatellite loci, enabling precise cell lineage phylogeny reconstruction [13]. This represents a significant improvement over previous techniques that limited detection to approximately 128 microsatellites per cell. Microsatellites - short, tandem DNA sequence repeats - can expand or contract due to replication slippage, with size variations serving as heritable markers for retrospective lineage tracing, even allowing tracking of repeated subclone divergences [4]. Microsatellite loci are typically neutral sequences but change rapidly during cell division, generating mutation rates of 10^-5^ to 10^-3^ mutations per locus per cell division - significantly higher than the approximately 10^-9^ mutations per SNP [2]. However, this high error rate presents technical challenges, as microsatellite capture and sequencing require DNA amplification prone to expansion/contraction errors that can mask existing mutations. Previous approaches using exponential whole genome amplification required low cell division rates to maintain accuracy.

The RETrace technique addresses these limitations by capturing both microsatellite information and DNA cytosine methylation status from the same cells, enabling simultaneous retrospective lineage tracing and cell type determination. By using linear amplification of microsatellites, RETrace reduces in vitro noise, producing higher-resolution and more accurate lineage trees while reliably categorizing cell types. The methylation status of approximately 150,000 distinct CpG sequences in microsatellites can be analyzed using single-cell reduced representation bisulfite sequencing to infer cell type based on chromatin methylation patterns.

Mitochondrial DNA (mtDNA) mutations as endogenous barcodes

Another valuable source of endogenous barcodes is mitochondrial heteroplasmy. Somatic mutations accumulate in mtDNA at rates 10 to 1,000 times higher than in nuclear DNA. Since most mtDNA mutations are noncoding and don't affect mitochondrial function, they pass to daughter cells during division. With hundreds of mitochondria (each containing multiple mtDNA copies) per cell, there are often over 10,000 mtDNA copies per cell, allowing substantial cell-to-cell sequence variation [14]. This enables the establishment of the cell lineage phylogenies with high sensitivity and specificity at the single-cell level [6].

The Epigenome and Mitochondrial Barcode of Lineage from Endogenous Mutations (EMBLEM) study simultaneously assessed cellular lineages using mtDNA mutations and cell fate via ATAC-seq, revealing genetic and epigenomic clonal evolution of acute myeloid leukemia from hematopoietic stem cells [6]. Similarly, combining mtDNA mutation lineage trees with ATAC-seq and scRNA-seq profiling has proven effective for studying clonal hematopoiesis by determining clonal origins, lineages, and cell states of each descendant [15-17]. These studies utilized protocols similar to dscATAC-seq or dsciATAC-seq, isolating individual cells in droplets containing Tn5 transposons that insert into open chromatin regions. The transposon-marked DNA was amplified, with mtDNA being enriched along with nuclear DNA. ATAC-seq naturally enriches mtDNA (which has an open structure) by 17-fold or more compared to whole genome sequencing, providing approximately 18,000 times greater mtDNA coverage with variable allele frequencies exceeding 90% for some regions [6]. This high-quality sequencing enabled the generation of cell lineage phylogenies based on mtDNA variants and mutations [6,17].

Beyond DNA sequence variations, naturally occurring alterations can also track lineages retrospectively. Pioneering work in the 1960s demonstrated cancer's clonal character by following X-chromosome inactivation, an epigenetic process [7]. Epigenetic changes like DNA methylation and hydroxymethylation can serve as lineage markers. Methyl groups added to cytosines in CpG dinucleotides are copied during DNA replication and inherited by daughter cells for multiple generations [4]. Since DNA methylation regulates gene expression, using CpG methylation as a barcode allows simultaneous determination of chromatin state as an indicator of cell fate. Temporal variations in methylation patterns make these ideal lineage tracing barcodes [4]. Other potential epigenetic barcodes include histone modifications, which are often copied into chromatin during cell division [4]. DNA hydroxymethylation offers unique tracking capabilities because it's not replicated equally - one daughter cell receives a highly hydroxymethylated strand, while the other gets a weakly hydroxymethylated strand, enabling differentiation between cells. Strand bias patterns of DNA hydroxymethylation have been used as lineage markers in embryonic mice, with differential hydroxymethylation between positive and negative strands precisely reproduced in subsequent divisions.

Limitations of retrospective lineage tracing

While retrospective lineage tracing offers numerous advantages, several limitations must be considered. The timing of endogenous mutation generation can significantly affect lineage tree structure, though phylogenetic errors can be reduced using maximum parsimony or complex tree reconstruction algorithms [7]. Maximum parsimony simplifies tree formation by minimizing the overall changes required for all lineages but ignores developmental periods between cell divisions except for mutation occurrences. Endogenous mutations used as barcodes may be infrequent and randomly distributed, potentially requiring whole genome sequencing that isn't always feasible. The low mutation rate can make finding suitable barcodes difficult [9].

Technical challenges include incomplete barcode detection and loss of barcoded RNA or chromatin fragments, resulting in missing lineage or cell state information. For example, in the original study by Raj et al. (2018), barcoded transcriptomes were only recaptured from 6% to 28% of cells in zebrafish embryos, meaning gene expression information for the remaining cells was lost [16]. Specifically for mtDNA-based lineage tracing, limitations include scarcity of mtDNA mutations in embryonic and young animal cells and tissues, which prevent the determination of lineage and cell state origination in early animal (and human) development; potential transfer of only a specific subset of mitochondria, and therefore of the mtDNA mutational barcodes, to daughter cells, such that barcodes are lost over time; and possibility of transfer of mitochondria between cells, resulting in the transmission of a barcode used to identify a specific progenitor cell to cells of a different lineage than the cells derived from the progenitor. However, in their study of droplet-based combinatorial indexing for chromatin accessibility, Macosko et al. found that asymmetric mitochondrial transmission and mitochondrial horizontal transfer were not significant issues for lineage tracing applications [15].

Additionally, using multi-omics to conduct lineage tracing of single-cells has the inherent limitation that certain cell types might be sparse in the sample, and therefore limit the statistical power of the analysis for those cell types, and thereby pose data resolution constraints by limiting the accuracy, and even the reliability and consistency of the data on these sparse cell types. In addition, retrospective methods also suffer from data resolution constraints that limit the ability to reconstruct detailed lineage paths. For example, noise in lineage traces, such as mutations not related to lineage (e.g., sequencing errors or random somatic changes), further complicate the reconstruction process. Finally, since the approach inherently analyzes the cells after they have reached terminal differentiation or are at least largely differentiated, dynamic processes that affect this differentiation cannot be tracked in real-time, requiring the use of prospective methods utilizing evolving barcoding, which are precisely intended for use in dynamic profiling, for certain types of analyses or studies.

Combining retrospective lineage tracing with multi-omic data and future directions

Standard lineage tracing determines cellular descent but cannot reveal the phenotypic state of each progeny. Single-cell multi-omics analyses combined with lineage information integrate complementary cell lineage and state data. Since retrospective lineage tracing already utilizes next-generation sequencing of endogenous DNA changes, it pairs well with other sequencing-based techniques. RNA-seq for single-cell transcriptome analysis can provide information on cell states, potential cell interactions, and more precise lineage determination. Combining retrospective lineage tracing with transcriptomics at single-cell resolution reveals differentiation trajectories and transcriptional alterations during lineage branching. Analyzing whole transcriptomes of barcoded cells via scRNA-seq techniques like Drop-seq (which isolates cells and barcodes in droplets) can provide information about cell cycle phase, metabolic state, and gene expression profiles to determine sub-lineages or identify new lineages.

Additional complementary techniques include chromatin conformation capture, chromatin accessibility detection, DNA methylomics, proteomics, metabolomics, or combinations thereof for individual cells [1]. Such analyses have revealed novel cell states, molecular signatures, and developmental transition states. Lineage tracing can also be combined with information on cell position, morphology, or gene expression to provide a more complete picture of clonally-derived cells. Recent spatial transcriptomics methods enable genomic measurements in situ, offering information on both cell state and position without previous limitations, and when used alongside lineage tracing, can determine how progenitor position affects lineage determination [11,15].

## Conclusions

Overall, as opposed to prospective lineage tracing, retrospective lineage tracing provides a way to track the development of cell types, tissues and organs, as well as cancers, that does not require prior access to the originating cells, nor the genetic manipulation of those cells that could affect their developmental progression in ways that could affect the very process under study. Thus, it is ideal for use in patients or patient-derived samples with limited availability, as well as in other cases involving the study of natural systems that are already in an intermediate or advanced stage of development. Furthermore, retrospective lineage tracing is also better suited to longitudinal studies of cell fate and lineage determination. The understanding of clonal growth of human tissues and cancers that retrospective lineage tracing provides will most probably lead to new therapies for a number of human diseases, and new uses for this approach will likely be discovered in the future.
